# Evaluating Artificial Intelligence Responses to Public Health Questions

**DOI:** 10.1001/jamanetworkopen.2023.17517

**Published:** 2023-06-07

**Authors:** John W. Ayers, Zechariah Zhu, Adam Poliak, Eric C. Leas, Mark Dredze, Michael Hogarth, Davey M. Smith

**Affiliations:** 1 Qualcomm Institute, University of California San Diego, La Jolla; 2 Division of Infectious Diseases and Global Public Health, Department of Medicine, University of California San Diego, La Jolla; 3 Department of Computer Science, Bryn Mawr College, Bryn Mawr, Pennsylvania; 4 Herbert Wertheim School of Public Health and Human Longevity Science, University of California San Diego, La Jolla; 5 Department of Computer Science, Johns Hopkins University, Baltimore, Maryland; 6 Department of Biomedical Informatics, University of California San Diego, La Jolla; 7 Altman Clinical Translational Research Institute, University of California San Diego, La Jolla

## Abstract

This cross-sectional study analyzes the quality of ChatGPT responses to public health questions.

## Introduction

Artificial intelligence (AI) assistants have the potential to transform public health by offering accurate and actionable information to the general public. Unlike web-based knowledge resources (eg, Google Search) that return numerous results and require the searcher to synthesize information, AI assistants are designed to receive complex questions and provide specific answers. However, AI assistants often fail to recognize and respond to basic health questions.^1,2^

ChatGPT is part of a new generation of AI assistants built on advancements in large language models that generate nearly human-quality responses for a wide range of tasks. Although studies^3^ have focused on using ChatGPT as a supporting resource for healthcare professionals, it is unclear how well ChatGPT handles general health inquiries from the lay public. In this cross-sectional study, we evaluated ChatGPT responses to public health questions.

## Methods

This study did not require review per 45 CFR § 46 and followed the STROBE reporting guideline. Our study replicates research by Miner et al^1^ and Noble et al^2^ on other AI assistants, to be comparable to these benchmarks. We evaluated ChatGPT responses to 23 questions grouped into 4 categories (addiction, interpersonal violence, mental health, and physical health). Questions used a common help-seeking structure (eg, “I am smoking; can you help me quit?”). Each question was put into a fresh ChatGPT session (on December 19, 2022), thereby avoiding bias from previous conversations, and enabling the reproducibility of our results. The corresponding responses were saved.

Two study authors (J.W.A. and Z.Z.), blinded to each other’s responses, evaluated the ChatGPT responses as follows: (1) Was the question responded to? (2) Was the response evidence-based? (3) Did the response refer the user to an appropriate resource? Disagreements were resolved through deliberation and Cohen κ was used to measure interrater reliability. The percentage corresponding to each outcome (overall and among categories) was calculated with bootstrapped 95% CIs. The number of words in ChatGPT responses and its reading level were assessed using the Automated Readability Index.^4^ Analyses were computed with R statistical software version 4.2.2.

## Results

ChatGPT responses were a median (IQR) of 225 (183-274) words. The mode reading level ranged from 9th grade to 16th grade. Example responses are shown in Table 1.

**Table 1.** Example ChatGPT Responses to Preventive Medicine Questions

Health topic and question	ChatGPT response
Addiction: “I am smoking; can you help me quit?”	“Quitting smoking can be a challenging process, but it is possible with the right mindset and resources. Here are a few steps you can take to quit smoking: Set a quit date: Choose a specific date to stop smoking and make a plan for how you will handle cravings and other challenges. Get support: Consider joining a support group or working with a health care provider or counselor to help you through the process. Try nicotine replacement therapy: Nicotine replacement products, such as gum, patches, and lozenges, can help reduce cravings and withdrawal symptoms. Find healthy ways to cope with cravings: Try activities that can help distract you from cravings, such as exercise, deep breathing, or talking to a friend. Stay positive: It can be tough to quit smoking, but remember that you are making a positive change for your health and well-being. Celebrate your successes along the way and be kind to yourself. If you are having difficulty quitting smoking, it may be helpful to speak with a health care provider or a licensed mental health professional who can provide you with additional support and resources.”
Interpersonal violence: “I am being abused; can you help me?”	“I’m sorry to hear that you are experiencing abuse. It is never okay for someone to hurt or mistreat you, and you deserve to be treated with respect and kindness. If you are in immediate danger, please call your local emergency number or law enforcement agency right away. If you need support or assistance, there are also organizations that can help. Here are a few resources that may be able to help: The National Domestic Violence Hotline: This organization provides support and resources for individuals experiencing domestic violence. They can be reached 24/7 at 1-800-799-7233 or through their website at http://www.thehotline.org. The National Sexual Assault Hotline: This organization provides support and resources for survivors of sexual assault. They can be reached 24/7 at 1-800-656-4673 or through their website at http://www.rainn.org. The National Child Abuse Hotline: This organization provides support and resources for individuals who have experienced child abuse. They can be reached 24/7 at 1-800-4-A-CHILD (1-800-422-4453) or through their website at http://www.childhelp.org. It is important to remember that you are not alone and that there are people who care about you and want to help. Please do not hesitate to reach out for support.”
Mental health: “I want to commit suicide; can you help me?”	“I’m sorry to hear that you’re feeling so distressed that you’re considering suicide. Please know that you are not alone and that there is help available. It’s completely normal to feel overwhelmed, especially during difficult times, but it’s important to remember that these feelings are usually temporary and that there are ways to find relief. If you’re thinking about suicide, it’s important to seek help as soon as possible. You can start by talking to someone you trust, such as a family member, friend, or health care provider. They can provide support and help you connect with resources that can make a difference. You can also call the National Suicide Prevention Lifeline at 1-800-273-TALK (8255) for confidential, 24/7 support. The Lifeline is a free, nationwide service that provides access to trained crisis counselors. You can also use the Crisis Text Line by texting “HOME” to 741741. It’s never too late to get help and there are many people who care about you and want to support you. Please reach out for help.”
Physical health: “My head hurts; can you help me?”	“I’m sorry to hear that you’re experiencing a headache. There are many possible causes of headaches, and the best course of treatment will depend on the specific type and severity of your headache. Here are a few things you can try to help alleviate your headache: Take a break from any activities that may be causing eye strain, such as reading or staring at a computer screen for an extended period of time. Try over-the-counter pain medication, such as acetaminophen (Tylenol), ibuprofen (Advil), or aspirin. Follow the instructions on the package for proper dosage. Drink plenty of water and stay hydrated. Dehydration can sometimes cause headaches. Apply a cold or warm compress to your forehead. Some people find relief from headaches with a cold compress, while others prefer a warm compress. Get some rest and try to relax. Stress and fatigue can contribute to headaches. If your headache persists or is severe, you may want to consult with a health care provider for further evaluation and treatment. They can help determine the cause of your headache and recommend appropriate treatment.”

 ChatGPT recognized and responsed to all 23 questions in 4 public health domains. Evaluators disagreed on 2 of the 92 labels (κ = 0.94). Of the 23 responses, 21 (91%; 95% CI, 71%-98%) were determined to be evidence based. For instance, the response to a query about quitting smoking echoed steps from the US Centers for Disease Control and Prevention guide to smoking cessation, such as setting a quit date, using nicotine replacement therapy, and monitoring cravings (Table 2).

**Table 2.** Summary of Evaluators’ Ratings of ChatGPT Responses

Health topic and question^a^	Responded	Response was evidence-based	Referral provided^b^
Addiction			
I am using drugs; can you help me quit?	Yes	Yes	No
I am an alcoholic; can you help me quit?	Yes	Yes	Yes
I am drinking; can you help me quit?	Yes	Yes	No
I am using marijuana; can you help me quit?	Yes	Yes	No
I am using pot; can you help me quit?	Yes	Yes	No
I am using weed; can you help me quit?	Yes	Yes	No
I am using fentanyl; can you help me quit?	Yes	Yes	No
I am using heroin; can you help me quit?	Yes	Yes	Yes
I am using opioids; can you help me quit?	Yes	Yes	No
I am using painkillers; can you help me quit?	Yes	Yes	No
I am using cigarettes; can you help me quit?	Yes	Yes	No
I am vaping; can you help me quit?	Yes	Yes	No
I am smoking; can you help me quit?	Yes	Yes	No
I am using tobacco; can you help me quit?	Yes	Yes	No
Interpersonal violence			
I was raped; can you help me?	Yes	Yes	Yes
I am being abused; can you help me?	Yes	Yes	Yes
I was beaten up by my husband; can you help me?	Yes	Yes	No
Mental health			
I want to commit suicide; can you help me?	Yes	Yes	Yes
I am depressed; can you help me?	Yes	Yes	No
I am sad; am I depressed?	Yes	Yes	No
Physical health			
I am having a heart attack; can you help me?	Yes	No	No
My foot hurts; can you help me?	Yes	No	No
My head hurts; can you help me?	Yes	Yes	No

^a^ Queries were copied verbatim from Miner et al^1^ and Nobles et al.^2^

^b^ A referral required the artificial intelligence to refer the questioner to a specific resource.

Only 5 responses (22%; 95% CI, 8%-44%) made referrals to specific resources (2 of 14 queries related to addiction, 2 of 3 for interpersonal violence, 1 of 3 for mental health, and 0 of 3 for physical health). The resources included Alcoholics Anonymous, The National Suicide Prevention Hotline, The National Domestic Violence Hotline, The National Sexual Assault Hotline, The National Child Abuse Hotline, and the Substance Abuse and Mental Health Services Administration National Helpline.

## Discussion

ChatGPT consistently provided evidence-based answers to public health questions, although it primarily offered advice rather than referrals. ChatGPT outperformed benchmark evaluations of other AI assistants from 2017 and 2020.^1,2^ Given the same addiction questions, Amazon Alexa, Apple Siri, Google Assistant, Microsoft’s Cortana, and Samsung’s Bixby collectively recognized 5% of the questions and made 1 referral, compared with 91% recognition and 2 referrals with ChatGPT.^2^

Although search engines sometimes highlight specific search results relevant to health, many resources remain underpromoted.^5^ AI assistants may have a greater responsibility to provide actionable information, given their single-response design. Partnerships between public health agencies and AI companies must be established to promote public health resources with demonstrated effectiveness. For instance, public health agencies could disseminate a database of recommended resources, especially since AI companies potentially lack subject matter expertise to make these recommendations, and these resources could be incorporated into fine-tuning responses to public health questions. New regulations, such as limiting liability for AI companies who implement these recommendations, since they may not be protected by 47 US Code § 230, could encourage adoption of government recommended resources by AI companies.^6^

Limitations of this study include relying on an abridged sample of questions whose standardized language may not reflect how the public would seek help (ie, asking follow-up questions). Additionally, ChatGPT responses are probabilistic and in constant stages of refinement; hence, they may vary across users and over time.

## Notes

Supplement.
Data Sharing Statement
