# Supplementary material for: Evaluating Artificial Intelligence Responses to Public Health Questions
Source: JAMA Netw Open. 2023 Jun 7;6(6):e2317517. doi: 10.1001/jamanetworkopen.2023.17517 (PMC10248742; doi:10.1001/jamanetworkopen.2023.17517)
Supplement: Supplement. — Data Sharing Statement [file jamanetwopen-e2317517-s001.pdf]

Data Sharing Statement

Ayers. Evaluating Artificial Intelligence Responses to Public Health Questions. *JAMA Netw Open*. Published June 07, 2023. doi:10.1001/jamanetworkopen.2023.17517

Data
Data available: Yes
Data types: Other (please specify)
Additional Information: The data are already in the ms.
How to access data: in the ms.
When available: With publication

Supporting Documents
Document types: None

Additional Information
Who can access the data: anyone
Types of analyses: ANy.
Mechanisms of data availability: just scrape table 2.
